# Far from being well understood: multiple protein phosphorylation events control cell differentiation in *Bacillus subtilis* at different levels

**DOI:** 10.3389/fmicb.2014.00704

**Published:** 2014-12-10

**Authors:** Jan Gerwig, Jörg Stülke

**Affiliations:** Department of General Microbiology, Institute for Microbiology and Genetics, University of GöttingenGöttingen, Germany

**Keywords:** biofilm formation, cross-talk, tyrosine phosphorylation, EpsB, PtkA

## Cell differentiation in *Bacillus subtilis*

In their endless struggle to survive in harsh and rapidly changing environments, many bacteria depend on their ability to live together as multicellular communities, also known as biofilms. In these communities cells are embedded within a self-produced slimy matrix that is mainly composed out of extracellular polysaccharides and proteins (Hall-Stoodley et al., 2004). This matrix enables the cells to cover a solid surface or to float as a community and can protect them from harmful environmental substances, such as antibiotics or competitors. In addition, biofilm or matrix production can also function as a virulence factor, as described for the genetic disorder cystic fibrosis that goes along with colonization by a *Pseudomonas aeruginosa* biofilm (Costerton et al., 1999). The Gram-positive soil bacterium *Bacillus subtilis* can choose between a variety of lifestyles such as sporulation, motility as an explorative lifestyle, biofilm formation, and the acquisition of genetic competence for the uptake of foreign DNA (López and Kolter, 2010).

In the *B. subtilis* biofilm communities, different groups of cells fulfill distinct functions, which are important for the well-being of the whole community of clonal identical bacteria. Some bacteria produce extracellular polysaccharides and proteins and thereby provide the matrix for the community. Other cells secrete exoproteases for degradation of protein as an alternative energy source (Marlow et al., 2014). Furthermore, cells within the biofilm can differentiate into spores when the community gets older and nutrients are limiting (López and Kolter, 2010). However, not all cells within a biofilm differentiate into spores upon nutrient limitation. Some members of a biofilm community can regain motility. This allows them to leave the biofilm and explore the environment for new sources of nutrients. From an evolutionary point of view the presence of different cell forms provides versatility and enables the bacterium to adapt rapidly to different environmental conditions. But how are these complex communities and the observed cell differentiation processes regulated?

## Novel regulatory tyrosine phosphorylation adds even more complexity to the regulatory network for cell differentiation

**Figure 1:**
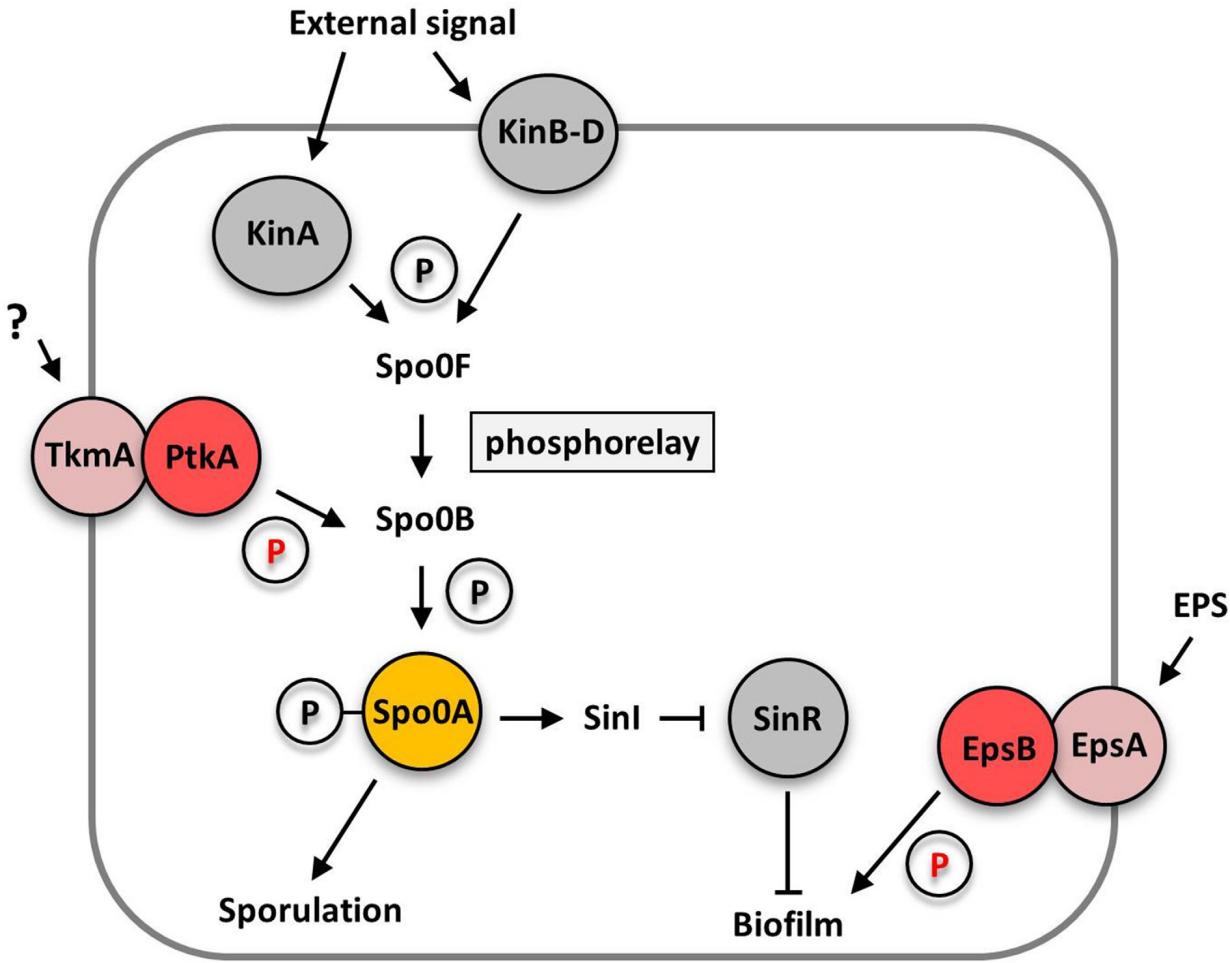
**Schematic overview how the bacterial tyrosine kinases PtkA and EpsB control cell differentiation at different levels**. The PtkA kinase controls sporulation and biofilm matrix expression by acting upstream of the central regulator of cell differentiation Spo0A by an unknown mechanism. In contrast, the EpsB kinase acts downstream of the Spo0A protein and controls exopolysaccharide production by phosphorylation of the glycosyltransferase EpsE. Arrows indicate activating effects, T-bars inhibitory effects. EPS, extracellular polysaccharides; P, phosphate group.

Recent studies in *B. subtilis* suggest that tyrosine phosphorylation plays an important role in the regulation of biofilm formation and cell differentiation, in addition to the known mechanisms of transcriptional regulation and protein-protein interactions (for review see Vlamakis et al., 2013; Mielich-Süss and Lopez, 2014; Mhatre et al., 2014). In Gram-positive bacteria, tyrosine kinases consist of a transmembrane modulator protein and a cytosolic kinase protein (Grangeasse et al., 2012). *B. subtilis* encodes two protein tyrosine kinase/ modulator couples, PtkA/ TkmA, and EpsB/ EpsA. Interestingly, the simultaneous deletion of either both kinase or modulator genes totally abolished extracellular polysaccharide production causing a biofilm defect. The single mutants did not phenocopy the kinase or modulator double mutant and were still able to produce exopolysaccharides. However, colony structure and pellicle formation was affected in the single mutants (Gerwig et al., 2014) suggesting that both kinase systems contribute in a distinct way to biofilm formation. The loss of the EpsB kinase reduced wrinkle formation and the production of extracellular polysaccharides, but did not destroy the rough colony surface, which is indicative of the formation of fruiting bodies for sporulation (Elsholz et al., 2014; Gerwig et al., 2014). Thus, EpsB does not seem to affect sporulation. In contrast, loss of the EpsB homolog PtkA did not affect extracellular polysaccharide production but instead drastically reduced sporulation in biofilm cells thus leading to a loss of the rough appearance of the outer region of the colonies (Kiley and Stanley-Wall, 2010; Gerwig et al., 2014). These observations indicate that the protein tyrosine kinases EpsB and PtkA influence cell differentiation of *B. subtilis* at different levels: EpsB acts downstream of the central regulator of cell differentiation, Spo0A, whereas PtkA is likely to act upstream of Spo0A (see Figure 1).

In principle, the stochastic phosphorylation state of the Spo0A protein determines to which promoters the protein binds and consequently if cells differentiate into a spore or become a matrix producer. High levels of phosphorylated Spo0A induce spore development, whereas medium levels lead to matrix production (Fujita and Losick, 2005; Fujita et al., 2005). Of course, Spo0A phosphorylation is highly regulated: it receives its phosphoryl groups via a complex phosphorelay system consisting of several sensor kinases and the Spo0F and Spo0B phosphotransferases. The phosphorelay is activated in response to multiple triggers such as the potassium concentration, plant polysaccharides and oxygen availability that are sensed by the Kin family sensor kinases (López et al., 2009; Beauregard et al., 2013; Kolodkin-Gal et al., 2013, see Figure 1). This highly complex regulatory network controlling the phosphorylation state of the central regulator of cell differentiation Spo0A allows the integration of many different signals into the phosphorelay. Furthermore, the phosphorelay provides multiple potential targets for post-translational control by the PtkA tyrosine kinase.

## How do the tyrosine kinases PtkA and EpsB influence cell differentiation?

In order to influence sporulation efficiency as shown by Kiley and Stanley-Wall (2010), PtkA most likely has to affect the phosphorelay that governs the phosphorylation state of the Spo0A protein. Since PtkA is a tyrosine kinase it seems likely that this influence involves post-translational tyrosine phosphorylation rather than acting e.g., on transcriptional level. Unfortunately, the most difficult question has not yet been solved: what is the phosphorylation target of the PtkA kinase and how can we identify it?

In order to explain altered biofilm formation and the sporulation defect of the *ptkA* mutant, Kiley and Stanley-Wall (2010) conducted an intensive search for possible phosphorylation targets but failed to identify one. Deletion of the long-known PtkA targets (the UDP-Glucose dehydrogenases Ugd and TuaD) did not exert an effect on biofilm formation. Moreover, several other targets proposed by large-scale phosphoproteomics and other studies (Macek et al., 2007; Jers et al., 2010) were not of relevance. Therefore, it remains unclear how PtkA affects biofilm formation and sporulation. Unfortunately, a recent phosphoproteome study did not reveal obvious targets related to the phosphorelay (Ravikumar et al., 2014), except the regulator of transition phase genes AbrB was found to be phosphorylated on a tyrosine residue. However, the physiological relevance of this phosphorylation is unclear, and serine phosphorylation of AbrB was observed in another study (Kobir et al., 2014). Clearly, more work is required to dissect the potential control of AbrB activity by phosphorylation.

A more obvious problem for the identification of tyrosine phosphorylated proteins with the potential to control biofilm formation and sporulation is that most published data relates to cells harvested from exponentially growing cultures rather than from biofilms. Moreover, the studies were performed with strains derived from a domesticated strain that does not produce robust bofilms. Thus, it is reasonable to assume that not all of the proteins that might be relevant for biofilm formation and sporulation are expressed under these conditions. Furthermore, regulatory phosphorylation is a rapid method for adapting cellular processes to environmental changes. Thus, it seems safe to assume that not all phosphorylations are present permanently. Interestingly, all currently identified tyrosine phosphorylation reactions with functional relevance have been found by attempts other than large-scale phosphoproteomics analyses. Examples include the regulator of unsaturated fatty acid synthesis FatR (Derouiche et al., 2013), single stranded DNA-binding proteins (Mijakovic et al., 2006) and the glycosyltransferase EpsE, a target of the tyrosine kinase EpsB (Elsholz et al., 2014). With the exception of UDP-glucose dehydrogenase Ugd (by Macek et al., 2007), none of these proteins were identified in the latest large-scale phosphoproteome experiments (Macek et al., 2007; Ravikumar et al., 2014). In conclusion, identification of the PtkA phosphorylation target and explanation of the sporulation defect of the mutant remains elusive but it is tempting to speculate that the highly complex network for the control of Spo0A is affected by the PtkA kinase. Since cross-phosphorylation of kinases is an established concept in eukaryotes and hints supporting this idea in prokaryotes are emerging (Baer et al., 2014; Shi et al., 2014) phosphorylation of phosphorelay proteins is a highly attractive hypothesis.

The second level of regulatory tyrosine phosphorylation is provided by the EpsB kinase that phosphorylates the glycosyltransferase EpsE (Elsholz et al., 2014). The kinase and the phosphorylation target are both encoded in the *eps* operon for exopolysaccharide production. Hence, the regulation of the two corresponding genes is similar. The *eps* operon is only strongly expressed if the SinR anti-activator protein is inhibited by either of its antagonists SinI and SlrR under biofilm forming conditions (Kearns et al., 2005; Newman et al., 2013; Winkelman et al., 2013). This observation implies that EpsB-mediated phosphorylation might not have a global effect and that the phosphorylated target is among the proteins expressed under biofilm forming conditions that are also subject to repression by SinR. Indeed, deletion of the *epsB* gene only affects exopolysaccharide production but leaves sporulation unaffected (Gerwig et al., 2014). Strikingly, deletion of the gene for the EpsE glycosyltransferase leads to a complete loss of exopolysaccharide production and complex colony formation, whereas deletion of the gene for the EpsB kinase has a milder effect (Guttenplan et al., 2010). Therefore, it is tempting to speculate that PtkA can partially take over the function of EpsB. However, this has not been demonstrated experimentally.

## Functional cross-talk between tyrosine kinase/ modulator couples

Straight signal transduction is an important issue for many conserved multi-component signal transduction system families and has been extensively studied for two-component regulatory systems and phosphotransferase system-controlled RNA-binding antitermination proteins. These systems have evolved to avoid non-cognate interactions either by restricting the interactions with non-cognate proteins partners, ligands, and target molecules. Moreover, differential gene expression of the non-cognate components has been observed to prevent non-productive cross-talk (Schilling et al., 2006; Szurmant and Hoch, 2010; Hübner et al., 2011; Podgornaia and Laub, 2013).

However, this might be different for regulatory tyrosine phosphorylation, as suggested for the interplay between EpsB and PtkA. In yeast (Shi et al., 2014) and bacterial two-hybrid studies the TkmA modulator and the EpsB kinase interact with each other, whereas the EpsA modulator and the PtkA kinase do not interact. Additionally, a genetic analysis of a potential cross-talk in the laboratory strain 168 revealed that simultaneous loss of PtkA and EpsA does not affect stable pellicle formation, whereas simultaneous deletion of the genes for EpsB and TkmA inhibited stable pellicle formation. These observations further support a functional connection between the two systems. However, confirmation of this result was not obtained in the background of the NCIB3610 wild type strain. Although the functional relevance of the TkmA/EpsB cross-talk remains unclear, similar observations come from *Staphylococcus aureus* that also contains two similar tyrosine kinase/ modulator couples. In this case, the Cap5A1 modulator protein of one couple and the Cap5B2 protein tyrosine kinase of the other couple show functional cross-talk suggesting that interplay between different tyrosine/ modulator couples might not be limited to *B. subtilis* (Soulat et al., 2007).

## Outlook

The detection of a regulatory interplay between protein tyrosine phosphorylation and classical sensing via the phosphorelay in the control of cell differentiation in *B. subtilis* is one of the most exciting results of recent studies. This is underlined by the observation of extensive links between the different signal transduction systems that involve post-translational modifications (van Noort et al., 2012; Shi et al., 2014) One main task for future work is the identification of phosphorylation targets of the tyrosine kinase PtkA in order to get a better understanding of its implication in biofilm formation and sporulation. To demonstrate that PtkA affects cell differentiation upstream of the central regulator Spo0A, the phosphorylation state of Spo0A has to be analyzed in a *ptkA* deletion mutant. Furthermore, large-scale phosphoproteomics under biofilm-promoting conditions could help to identify potential tyrosine phosphorylated targets. Additional tasks are the identification of substances that can be sensed by the PtkA modulator protein TkmA and to further dissect the potential cross-talk between the two known tyrosine kinase/ modulator couples EpsB/ EpsA and PtkA/ TkmA in *B. subtilis*.

### Conflict of interest statement

The authors declare that the research was conducted in the absence of any commercial or financial relationships that could be construed as a potential conflict of interest.
